# The Phospholipid Molecular Species Profile of *Apostichopus japonicus* Tissues Modifies through Exposure to n-3 Polyunsaturated Fatty Acid-Deficient Diet

**DOI:** 10.3390/md20090578

**Published:** 2022-09-15

**Authors:** Ekaterina V. Ermolenko, Tatyana V. Sikorskaya, Valeria P. Grigorchuk

**Affiliations:** 1 A.V. Zhirmunsky National Scientific Center of Marine Biology, Far Eastern Branch, Russian Academy of Sciences, ul. Palchevskogo 17, 690041 Vladivostok, Russia; 2 Federal Scientific Center of the East Asia Terrestrial Biodiversity, Far Eastern Branch, Russian Academy of Sciences, Pr-t 100-let Vladivostoka 159, 690022 Vladivostok, Russia

**Keywords:** sea cucumber, *Apostichopus japonicus*, aquaculture, lipidomics, phospholipids

## Abstract

The sea cucumber *Apostichopus japonicus*, being a target species of commercial fisheries and aquaculture, is also used as a source of biologically active compounds with high pharmacological potential. By the methods of high-performance liquid chromatography with high resolution mass spectrometry, we analyzed the major structural phospholipids (PL)—glycerophosphoethanolamines (PE), glycerophosphocholines (PC), glycerophosphoserines (PS), and glycerophosphoinositols (PI)—in tissues of wild and cultured sea cucumbers. The intestines of the wild and cultured animals differed from the other tissues by an elevated content of molecular species of PE, PC, and PS with 22:6n-3 fatty acid. The respiratory trees of the studied animals contained a high level of odd-chain PI and PI with 20:4n-6. The exposure to n-3 PUFA-deficient diet resulted in substantial changes in the molecular species profile of PL of the wild and cultured animals. The cultured sea cucumbers showed a significant decrease in the 20:5n-3 content in all four studied PL classes. A replacement of 20:5n-3 by 20:4n-6 occurred in PE, PC, and PI. The decrease in the level of molecular species of PS with 20:5n-3 was compensated by an increase in the level of monounsaturated long-chain PS. The diet of cultured sea cucumbers is a crucial factor for enhancing the nutritional properties of the product obtained from them.

## 1. Introduction

Sea cucumbers or holothurians (Echinodermata: Holothuroidea) are important members of benthic invertebrate communities found across the world’s oceans and major seas. Feeding on detritus and organic matter, they play an important role in marine ecosystems [1,2]. The sea cucumber *Apostichopus japonicus* is distributed coastal waters off the Korean Peninsula, Japan, China, and Russia within latitudes from 35° to 44° N [1]. *A. japonicus* is a target species of commercial fisheries and aquaculture, with the total annual production in China only reaching 204,359 t in 2017 [3]. It is a source of biologically active compounds with high pharmacological potential such as polysaccharides (sulfated fucans and fucosylated chondroitin sulfates), triterpene glycosides (holotoxins A, B, D, E, F, G, and H), cerebrosides, and essential fatty acids (FA) [4]. The range of biological activities of the substances isolated from *A. japonicus* includes the anticoagulant, antithrombotic [5], antifungal [6], and antihyperlipidemic [7] activity, and also the immunity stimulating effect [8].

Lipids as a non-protein energy source play an important role in providing energy and essential fatty acid for normal growth and survival of aquatic animals. Long-chain (C_20–22_) polyunsaturated fatty acids (PUFA, especially arachidonic (ARA, 20:4n-6), eicosapentaenoic (EPA, 20:5n-6), and docosahexaenoic (DHA, 22:6n-6) acids) as essential components of biomembranes of all cells and tissues providing the normal growth and ontogeny [9]. Polar lipids (glycerophospholipids, sphingolipids, and glycolipids) are structural components of cell membranes involved in signal transduction. *A. japonicus* contains about nine major classes of lipids, of which polar lipids are represented by cerebrosides, glycerophosphoethanolamines (PE), glycerophosphocholines (PC), glycerophosphoserines (PS), and glycerophosphoinositols (PI) [10]. The cerebrosides from *A. japonicus* was identified earlier [11]. Among phospholipids, the composition of PE and PC in this holothurian was studied in sufficient details [12].

The effect of diet on growth, survival, and lipid composition of cultured *A. japonicus* has been intensively studied [13,14,15,16,17]. The importance of n-3 PUFA for growth of *A. japonicus* has been shown in works [14]. The high content of n-6 PUFA in the diet of cultured sea cucumbers leads to significant differences in the FA composition between wild and cultured sea cucumbers, of which the major one is the trace amounts of 16:1n-7, a considerably higher level of ARA, and a lower level of EPA for cultured animals [10,18].

The current advance of analytical techniques allows collecting much more extensive arrays of data on lipid compositions and, in particular, profiles of lipid molecular species (distribution and *sn*-position in the glycerol backbone of acyl, alkyl, and alkenyl groups). The knowledge of lipid molecular species composition can be used to study lipid biosynthesis pathways, chemotaxonomy, determine biotic and abiotic factors effects, investigate embryogenesis, ontogenesis, and food chains [19,20]. Our work aimed to analyze the molecular species composition of the major structural phospholipids (PC, PE, PS, and PI) in tissues of wild and cultured sea cucumbers, *A. japonicus*, fed a diet deficient in n-3 PUFA.

## 2. Results

MS2 fragmentation allows characterizing the composition of phospholipid molecular species. For the wild animals, 38, 47, 31, and 41 molecular species of PE, PC, PI, and PS, respectively, were present in the lipids (Table S1). The cultured animals contained 38, 43, 27, and 40 molecular species of PE, PC, PI, and PS, respectively (Table S1).

**Figure 1:**
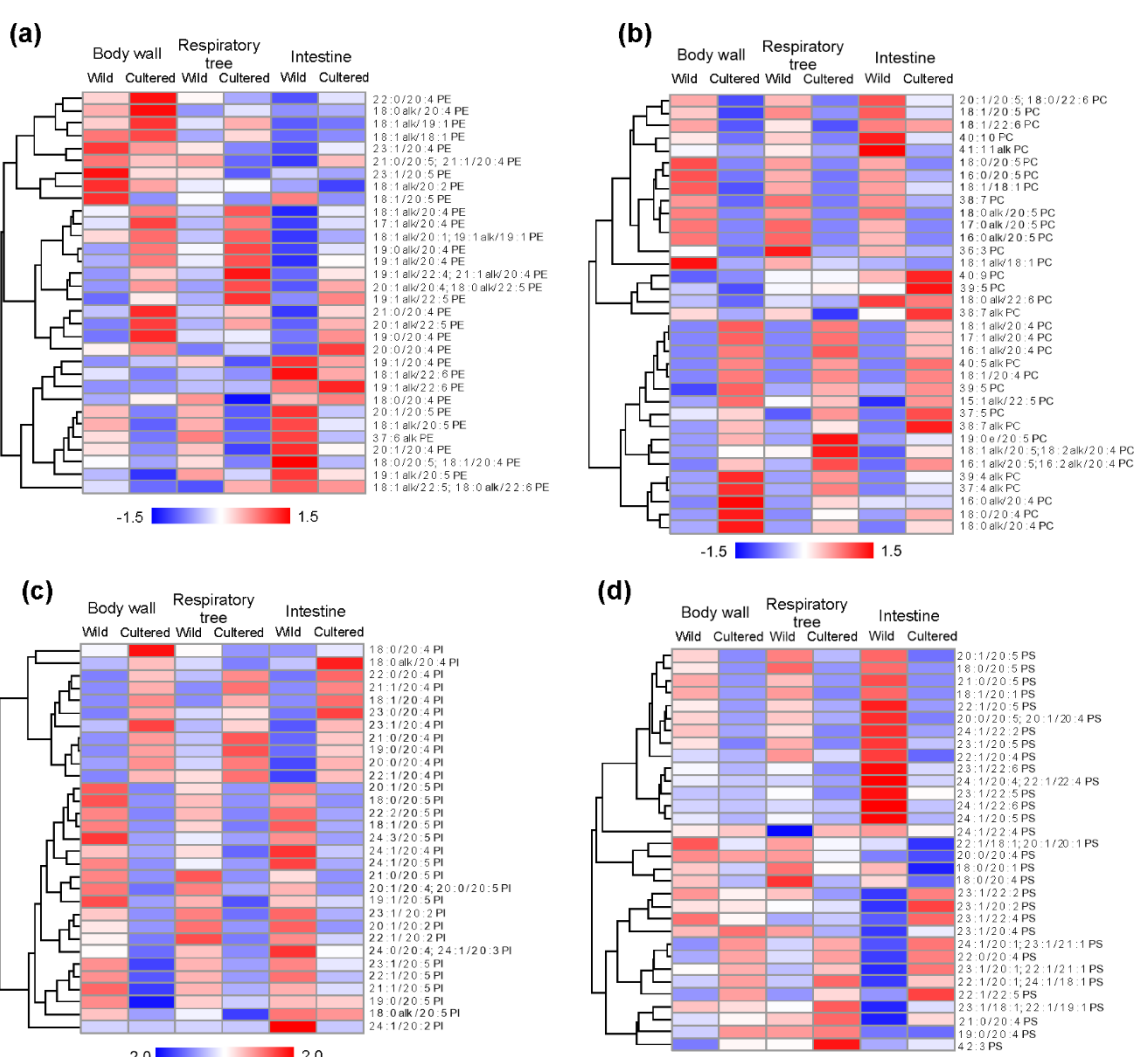
Heat maps of average data of phospholipid molecular species with a clustering (tree clustering, wards method, and Euclidean distances): (**a**) glycerophosphoethanolamines (PE), (**b**) glycerophosphocholines (PC), (**c**) glycerophosphoinositols (PI), and (**d**) glycerophosphoserines (PS). The scale bar above the heatmap(s) represents the standard scaling to the relative abundance of lipid content (% of each class) in the samples.

A heat map of lipids was used to visualize the phospholipid profiles of all the studied samples (body wall, respiratory tree (RT) and intestine of wild and cultured of sea cucumbers (Figure 1). For heat map creation we used the content of PL molecular species (average data of % of each phospholipid class) based on the results of a two-way ANOVA and HSD test (*p* < 0.05) (Table S1).

The PE consisted primary of EPA and ARA in all the samples studied (Figure 1a, Table S1). The alkyl fragment was present at the *sn*-1 position of glycerol in 82.5% and 87.6% of total PE (average of tissues) for the wild and cultured animals, respectively (Table 1). The major PE molecular species with a content of more than 5% of total PE in the studied samples were 18:1alk/20:5 PE, 18:1alk/20:4 PE, 19:1alk/20:5 PE, 19:1alk/20:4 PE, 18:1alk/22:5; 18:0alk/22:6 PE, and 20:1alk/20:4; 18:0alk/22:5 PE. The cultured sea cucumbers were distinguished by a higher level of PE with ARA in their composition (HSD test, *p* < 0.05) for all tissues (Table 1). The PE composition of RT from cultured sea cucumbers contained a high amount of alkyl PE (92.8% of total PE) compared to the body wall and intestine (85.5% and 84.5% respectively). The intestines of both the wild and cultured samples were characterized by an elevated content of PE molecular species with EPA and DHA in their compositions.

The PC composition was represented as heat maps and supplementary materials (Figure 1b, Table S1). The major PC molecular species in the studied samples were 18:1alk/20:5;18:2alk/20:4 PC, 18:0alk/20:5 PC, 18:1alk/20:4 PC, 18:0alk/20:4 PC, 18:1/20:5 PC, 18:0/20:5 PC, 18:1/20:4 PC, 18:1/22:6 PC, and 20:1/20:5;18:0/22:6 PC (Table S1). Significant differences between the tissue types were not found both for the wild and cultured animals. The wild animals contained about 42.0% alkyl PC in all tissues. Lipids of the cultured specimens differed by a higher level of alkyl PC compared to the wild sea cucumbers (HSD test, *p* < 0.05) (Table 1). The cultured specimens contained a different amount of alkyl PC. The body wall differed by a higher alkyl PC content (70.8% alkyl PC of total PC) from RT (66.7% of total PC) and intestine (55.1% of total PC) (HSD test, *p* < 0.05). Similarly to PE, all the tissue samples from the cultured sea cucumbers were distinguished by a higher level of PC with ARA (HSD test, *p* < 0.05) (Table 1). The higher content of molecular species with EPA was shown for lipids of the RT and intestine of the cultured animals compared to the body wall (HSD test, *p* < 0.05). The content of PC molecular species with DHA in the intestines of the wild and cultured samples was higher compared to the body wall and RT.

The PI composition was represented mainly by the following molecular species: 20:1/20:5 PI, 20:1/20:4;20:0/20:5 PI, 20:0/20:4 PI, 22:1/20:5 PI, 22:1/20:4 PI, 23:1/20:5 PI, 23:1/20:4 PI, 24:1/20:5 PI, and 24:1/20:4 PI (Figure 1c, Table S1). Only two PI molecular species with ether bond were found (18:0alk/20:5 PI and 18:0alk/20:4 PI). The PI composition included odd-chain FA: 19:0, 19:1, 21:0, 21:1, 23:0, and 23:1. The cultured sea cucumbers contained a higher level (% of total PI) of molecular species with ARA and odd-chain FA (HSD test, *p* < 0.05) (Table 1). A comparison of the tissue types in the wild animals showed that RT differed from the body wall and intestine by an elevated amount of PI with ARA and odd-chain FA (HSD test, *p* < 0.05); the intestine contained a higher level of long-chain FA (more than 42 carbon atoms in acyl fragments) in PI. The intestine of the cultured animals contained 13.9% of total PI molecular species with EPA; the RT and body wall contained 9.2% of total PI and 6.4% of total PI, respectively (Table 1). The RT of cultured animals contained a lower level (25.3% of total PI) of long-chain molecular species compared to the intestine and body wall (31.5% and 35.5% of total PI respectively). A lower level of odd-chain FA in the PI composition in the intestine (HSD test, *p* < 0.05) compared to the body wall and RT was shown for the cultured animals.

PS differed from the other PL classes by its high content of long-chain FA (more than 75.6% of total PS). The major FA in PS were 20:1, 23:1, 22:1 and, 24:1 (Figure 1d, Table S1). All the tissue samples from the cultured sea cucumbers were distinguished by a lower level of PS with eicosapentaenoic acid expressed in terms of % of total PS (HSD test, *p* < 0.05). No statistically significant differences were found in the content of molecular species with ARA. All the tissues of the cultured animals contained a high level of long-chain FA in PS compared to those of the wild specimens (HSD test, *p* < 0.05). A comparison of the tissue types of the wild animals showed that intestine PS contained a high level of EPA and DHA, but a lower level of molecular species with ARA (Table 1). The odd-chain molecular species of PS decreased in a body walls, RT, and intestines of the wild animals (Table 1). The intestines of the cultured animals contained a high level of PS molecular species with DHA, odd-chain and long-chain FA.

**Figure 2:**
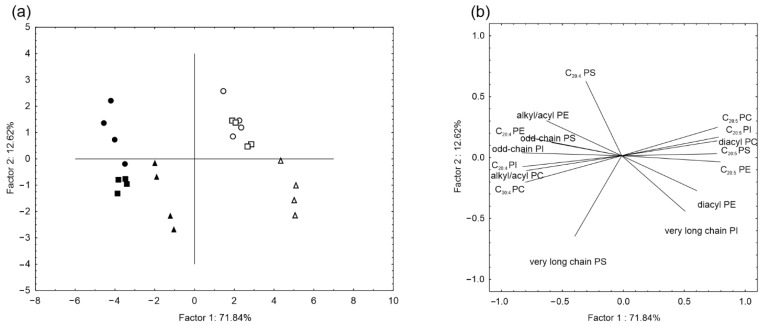
Results of principal components analyses (PCA) on the basis of lipid molecular species composition (% of each lipid class) of the body wall, respiratory tree and alimentary canal of wild and cultured *Apostichopus japonicus* sea cucumbers. Symbols are as follows: ● respiratory tree, ■ body wall and ▲ intestine of cultured specimens; and ○ respiratory tree □ body wall and ∆ intestine of wild specimens. PE: glycerophosphoethanolamines; PC: glycerophosphocholines, PI: glycerophosphoinositols; PS: glycerophosphoserines.

The main differences between tissues of the wild and cultured animals are presented in Table 1. The intestines of the wild and cultured animals differed from the other tissues by an elevated content of molecular species of PE, PC, and PS with DHA. The RT of the wild and cultured animals contained a high amount of odd-chain PI and PI with ARA. Figure 2a shows that all cultured samples are located separately from all wild samples along the first PCA component, linking positively with EPA containing PL (r = 0.96 for C20:5 PI, r = 0.94 for C20:5 PS, r = 0.98 for C20:5 PE, r = 0.95 for C20:5 PC) diacyl molecular species of PC (r = 0.95) and PE (r = 0.74), and very long chain PI (r = 0.63) (Figure 2b). The negative link of first component was showed for ARA containing PL (r= −0.98 for C20:5 PI, r= −0.35 for C20:5 PS, r= −0.93 for C20:5 PE, r= −0.95 for C20:5 PC), alkyl/acyl PE (r= −0.74) and PC (r= −0.95), odd chain PI (r= −0.99) and PS (r= −0.69), and very long chain PS (r= −0.46).

The pairwise comparison based on one-way ANOVA and HSD test (*p* < 0.05) showed that the most of PL molecular species significantly different between wild and cultured sea cucumbers (Table S2). The cultured sea cucumbers were characterized by a significant decrease in the EPA content in all four studied PL classes. For PE, PC, and PI, an increase in ARA levels was observed in the cultured samples. For PS, a decrease in molecular species from EPA resulted in an increase in long-chain monounsaturated PS (23:1/20:1;22:1/21:1 PS, 24:1/20:1; 23:1/21:1 PS, and 22:1/20:1;24:1/18:1 PS). The cultivated animals differed from the wild ones in an elevated content of alkyl PC and odd-chain PI.

## 3. Discussion

The advance in the chromatographic and mass spectrometric methods of analysis has made it possible to identify and quantify all molecular species of lipids in marine invertebrates [20]. Previously, the lipidomic approach was used to analyze phospholipids of six edible holothurians: *Parastichopus califormicus*, *Cucumaria frondosa*, *Isostichopus fuscus*, *Holothuria mexicana*, *Holothuria polli*, and *Bohadschia marmorata* [21]. A heatmap of the profile of lipid molecular species showed that different species of sea cucumbers were grouped by branch clustering, which could be helpful for detecting similar nutrients or avoiding misidentification of different dried sea cucumbers [21]. The composition of PE and PC with determination of *sn*-1/*sn*-2 position of alkyl or acyl fragments in *A. japonicus* was shown earlier [12]. We compared the profiles of molecular species of the major phospholipid classes in three types of tissues of wild and cultured *A. japonicus* sea cucumbers. Generally, our results of phospholipid composition were consistent with published works [12,21]. A high PUFA content was found in PC, PE and PI; very-long chain and odd-chain FA were detected predominantly in PS and PI. These are common features of holothurian phospholipids.

### 3.1. Phospholipid Molecular Species Profile in Tissues of A. japonicus

The composition of lipid classes and fatty acids in different tissues was determined earlier [10,12,18,22]. We considered the parameters of phospholipid composition common to wild and cultivated animals. Our data showed that the intestines of wild and cultured animals differed from the other tissues by an elevated content of molecular species of PE, PC, and PS with 22:6 FA. Similar results for the FA composition of intestine were obtained earlier [10,18]. The role of DHA in phospholipid composition of epithelial cells has been shown for gilthead seabream [23]. The authors reported that DHA deficiency dramatically altered the membrane lipid composition in enterocytes and strongly affected the thermodynamic properties of epithelial Na+/K+-ATPase in vivo. Echinoderms are known as typical osmoconformers. A good capacity for maintaining the intestinal cell volume under osmotic challenges (especially hyper-osmotic) with the involvement of Na+-K+-ATPase and Na-K-Cl cotransporter has been shown for *Holothuria grisea* [24].

The RT of the wild and cultured animals contained a high amount of odd-chain PI and PI with 20:4 FA. In holothurians, the RT is an organ involved in gas exchange, elimination of metabolic by-products, and maintenance of celomic fluid turgor [25]. One of the studies that evaluated the activity of digestive enzymes in the RT and digestive tract has shown that *Isostichopus badionotus* seems to be able to digest foods directly from the water through the RT, maximizing the use of available nutrients by luminal, intracellular, and apical membrane-linked digestive modes [26]. Such diverse RT functions are likely to be regulated by numerous cellular mediators such as eicosanoids. The role of the PI molecular species with ARA in holothurian RT is not entirely understood to date. It is possible that PI act as precursors of eicosanoids and phosphoinositides that have crucial roles in interfacial binding of proteins and in the regulation of protein activity at the cell interface [27].

The body wall is the edible part of sea cucumber, consisting of mutable collagenous tissue [28]. No common features of body wall phospholipid compositions were found in wild and cultured sea cucumbers, with the differences between them being very significant. The differences between the body wall and other tissues were shown for some minor molecular species of PE and PS.

### 3.2. Differences in Lipid Profile Composition between Wild and Cultured Sea Cucumbers

Previously, a comparison of lipid and fatty acid compositions between wild and cultured sea cucumbers showed significant differences [18]. The authors reported that the exposure to deficiency of dietary PUFA during cultivation considerably reduces the level of total and storage lipids in the alimentary canal, modifies the FA composition of all tissues, and increases the difference in the FA profile between the body wall and the alimentary canal [10]. Our work identified the phospholipid classes are most subject to changes in their composition at dietary deficiency of n-3 PUFA. The content of n-3 PUFA was similar to that reported in the work [10] and amounted to 1.1–1.3% of total FA.

The n-3 PUFA deficiency to a great extent affected the molecular species composition of all studied phospholipids in tissues of the wild and cultured animals. We detected a decline in the EPA content in the phospholipids under study. Similar results in total lipids were obtained for *A. japonicus* body wall and alimentary canal earlier [10,18]. A replacement of EPA by ARA occurred in PE, PC, and PI. The decrease in the level of PS molecular species with EPA was compensated by an increase in the level of monounsaturated long-chain PS. The effect of dietary PUFA levels on the expression of fatty acid elongase 5 (AJELOVL5), the PUFA composition, and the growth in juvenile *A. japonicus* were studied earlier [17]. Authors reported that the dietary balance in n-3 PUFA content was a key factor affecting the AJELOVL5 expression and growth of sea cucumbers [17]. The effect of diet with n-3 PUFA on growth and body biochemical composition of juvenile sea cucumbers *I. badionotus* [29] and *A. japonicus* [30] was studied earlier. The optimum level of lipids with a high n-3 PUFA content was determined in a study on *A. japonicus* [30]. Authors propose diets with higher levels of PUFA, in particular a high DHA content, as one of the best options to increase growth of these organisms [29].

Such significant modifications in the phospholipid composition of tissues can affect the growth and survival of animals. The importance of PL for growth, development, and survival has been shown for teleost fish predominantly [31]. In addition to the structural and energy functions, phospholipids play a crucial role in the regulation of metabolism and physiology as precursors for a range of highly biologically active mediators of metabolism and physiology including eicosanoids, diacylglycerol, inositol phosphates, and platelet activating factors [31]. Disruption of the EPA and ARA ratios can induce various disturbances affecting growth, development, and survival of sea cucumbers in aquaculture conditions. A change in the ARA and EPA ratio in the phospholipid composition can contribute to the imbalance of eicosanoids formed from ARA or EPA. As known, the products of ARA and EPA oxidation with the involvement of lipoxygenase and cyclooxygenase have different activities. ARA acts as a precursor for mediators responsible for inflammation, while eicosanoids from EPA have anti-inflammatory properties [32]. In Echinodermata, the lipoxygenase products are known to play roles in oocyte maturation in starfish and prevention of polyspermy in sea urchins [33]. In mammals, leukotriene B4 stimulates phagocytosis, chemotaxis, and aggregation in neutrophils, and the release of lysozyme and generation of oxygen radicals [34]. However, such activity of lipoxygenase products in *Asterias rubens* has not been confirmed to date [35]. The role of eicosanoids in holothurians needs further research.

In our work, we observed interesting features related to alkyl/acyl form of PC. All the studied tissues of the cultured sea cucumbers contained a higher level of alkyl/acyl PC compared to the wild animals’ tissues. According to previously published data, PC in alkyl/acyl form are characteristic of body wall lipids in *A. japonicus* [12]. The increase in the amount of alkyl molecular species in one of the major phospholipids is probably a compensatory mechanism for elevated ARA concentration. The different family members of phospholipase A2 demonstrated substrate specificity to diacyl, alkyl/acyl, or alkenyl/acyl form of PL [36]. The alkyl/acyl form of PC may protect ARA from enzymatic oxidation, as ARA are more preferred to lipoxygenase action [37].

## 4. Materials and Methods

### 4.1. Sample Collection

Wild specimens of the sea cucumber *A. japonicus* (Selenka, 1867) aged 1 yr (weighing 12–16 g) were collected from Peter the Great Bay (Sea of Japan) in February 2021 (42°58′58″ N, 131°45′11″ E). Cultured specimens (weighing 11–13 g and aged 11 month) were from the same locality: they were reared at the Scientific and Experimental Mariculture Hatchery, NSCMB FEB RAS, and kindly provided by S.I. Maslennikov in the same season. The cultivation conditions were similar and are described in detail in the work [10]. Sea cucumbers were cultured in 200 L fiberglass tanks and fed with a daily ration of 5% of their wet body weights. The water salinity ranged from 28‰ to 30‰; aeration was provided continuously. The diet of cultured animals consisted from a mixture of dry benthic silt (95%) and a compound diet for juvenile fish (5%) (China).

Four wild and four cultured specimens were selected for lipid analysis. The body of each specimen was dissected along the right ventral interradius. The respiratory tree (RT) and intestine were removed under sterile conditions. The intestine was dissected along its length to remove contents and then rinsed with sea water. Total lipids were immediately extracted from the freshly dissected tissues.

### 4.2. The Lipid Analysis

PL standards, including phosphatidylcholine (12:0/12:0 PC), phosphatidylethanolamine (18:0/18:1 PE), phosphatidylserine PS (16:0/16:0), and phosphatidylinositol (18:0/18:0 PI) were purchased from Avanti Polar Lipids, Inc. (Alabaster, Al, USA). Isopropanol and n-hexane (MS grade) were purchased from Honeywell Riedel-de Haen (St. Louis, MO, USA). Ammonia solution, triethylamine, and formic acid were purchased from Sigma-Aldrich (St. Louis, MO, USA). The ultrapure water was obtained using a Milli-Q water purification system (Millipore, MA, USA). Chloroform and methanol (analytical grade) were purchased from Vekton (St. Petersburg, Russia).

Total lipids were extracted with a chloroform/methanol mixture according to [38], evaporated under reduced pressure, weighed, dissolved in chloroform and stored at −40 °C. To analyze the content and structure of the molecular species of phosphorus-containing lipids, the total lipids were separated on a Shim-Pack diol column (4.6 mm × 50 mm, particle size 5 μm) (Shimadzu, Japan) using a Nexera-e chromatography system (Shimadzu, Japan). Solvent system A (2-propanol:hexane: H_2_O:HCOOH:28% NH_4_OH:Et_3_N, 28:72:1.5:0.1:0.05:0.02, *v*/*v*) and solvent system B (2-propanol:H_2_O:HCOOH:28% NH4OH:Et_3_N, 100:1.5:0.1:0.05:0.02, *v*/*v*) were used as eluents. System B content was programmed as follows: 0% (8 min), 0 to 20% (7 min), 20 to 100% (5 min), 100% (15 min), 100 to 0% (0.1 min), and 0% (12 min). The elution rate was 0.2 mL/min. To detect lipids, a high-resolution tandem mass spectrometer LCMS-IT-TOF (Shimadzu, Japan) was used. Analysis was performed under the electro-spray ionization (ESI) mode with simultaneous registration of signals of positive and negative ions. Scanning was performed in a *m*/*z* range of 100–1400. Source voltage was −3.5 kV in case of negative ion formation and 4.5 kV in case of formation of positive ions. The temperature of the ion source was 200 °C; dry gas (N2) pressure, 150 kPa; the flow rate of nebulizing gas (N2), 1.5 L/min. Argon (0.003 Pa) was used in the collision chamber of the mass spectrometer. The structural identification of each lipid molecular species was conducted by LC-MS analysis; this entailed comparing the retention times, ion forms, and specific fragmentation behaviors of the phospholipid classes with the lipid standards that commercially available. The detailed information of identification described earlier [39,40]. Percentages of the individual molecular species of each lipid class were calculated by peak area of negative ions [M–H]^−^, except for PC that was estimated peak area of negative ions [M+HCOOH]^−^.

### 4.3. Statistical Analysis

The raw data obtained were tested for the homogeneity of variances (Levene’s test) and normality of data distribution (Shapiro–Wilk’s test). Differences in the mean concentration of PL molecular species (% of each PL class) were analyzed by one–way and two-way analysis of variance (ANOVA). The factors were the origin (wild and cultured) and the tissue (body wall, RT, and intestine). Both factors were fixed. The significant differences between the levels within the factors were assessed post hoc using the Tukey’s HSD test. The preliminary data was arcsine-transformed prior to the principal component analysis (PCA). All statistical analyses were performed using the STATISTICA 12 package (StatSoft, Inc., Tulsa, OK, USA). A probability level of *p* < 0.05 was considered statistically significant. Values are presented as the mean ± standard deviation. To represent differences between samples, heat maps were built using the R statistical software. The number of specimens (4 samples of each type tissue (respiratory tree, body wall and intestine) of wild and cultured animal) taken for the experiment is due to the complexity of the analysis to identify the molecular species of phospholipids.

## 5. Conclusions

N-3 PUFA deficiency leads to significant changes in the molecular species profile of phospholipids. The effect of the EPA decrease in phospholipids on the vital activity of sea cucumbers requires further studies. However, as was previously shown, dietary n-3 PUFA lead to an increase in the growth rate of juvenile *A. japonicus* [17,30]. The composition of the obtained products has a great implication for aquaculture. The importance of n-3/n-6 PUFA balance in the human diet has been shown in several reviews [32,41] and is especially important for inflammation. PUFA acts on inflammation through the oxylipins production and the regulation of transcription factors and epigenetic changes [41]. Evidence for the anti-inflammatory effect of n-3 PUFA on the immune response was shown in human studies [42,43]. Currently, special attention is paid to the implication of n-3 PUFA-containing phospholipids in the human diet, in particular, that aimed at maintaining brain health [44,45]. Therefore, the diet is a crucial factor both for the successful development and growth of cultured sea cucumbers and for enhancing the nutritional properties of the products obtained from them.

## Figures and Tables

**Table 1:** Contents of the major PL groups (% of total molecular species for each PL class, mean ± SD, *n* = 4) in the body wall, respiratory tree (RT), and intestine of wild and cultured sea cucumbers, *Apostichopus japonicus*.

PL Groups	Wild			Cultured		
	Body Wall	RT	Intestine	Body Wall	RT	Intestine
Phosphatidylethanolamines (% from total PE)						
C_20:5_ PE	30.62 ± 1.26 ^a^	31.39 ± 1.61 ^a^	42.30 ± 1.74 ^a^	11.02 ± 0.67 ^b^	10.41 ± 0.39 ^b^	22.64 ± 4.19 ^b^
C_20:4_ PE	43.85 ± 1.54 ^a^	44.75 ± 3.22 ^a^	27.15 ± 0.93 ^c^	62.45 ± 0.54 ^b^	57.40 ± 2.78 ^b^	48.12 ± 4.00 ^c^
Alkyl/acyl PE	83.76 ± 2.10 ^a^	82.30 ± 1.65 ^a^	81.30 ± 2.29 ^a^	85.46 ± 0.18 ^a^	92.80 ± 1.05 ^b^	84.46 ± 1.54 ^a^
C_22:6_ PE	10.02 ± 1.21 ^a^	8.11 ± 1.10 ^a^	16.32 ± 1.11 ^b^	8.26 ± 0.58 ^a^	9.68 ± 1.91 ^a^	13.38 ± 0.86 ^c^
Phosphatidylcholines (% from total PC)						
C_20:5_ PC	46.82 ± 0.90 ^a^	44.36 ± 0.82 ^a^	46.89 ± 1.01 ^a^	8.76 ± 0.97 ^b^	13.23 ± 1.81 ^c^	16.61 ± 1.15 ^d^
C_20:4_ PC	2.86 ± 0.19 ^a^	2.81 ± 0.45 ^a^	2.56 ± 0.20 ^a^	47.35 ± 0.88 ^b^	42.31 ± 1.31 ^c^	31.60 ± 2.11 ^d^
Alkyl/acyl PC	42.62 ± 2.68 ^a^	44.42 ± 3.33 ^a^	41.63 ± 1.24 ^a^	70.76 ± 0.94 ^b^	66.66 ± 1.91 ^b^	55.12 ± 0.77 ^c^
C_22:6_ PC	12.49 ± 0.41 ^a^	11.61 ± 1.46 ^a^	15.78 ± 0.42 ^b^	7.15 ± 0.60 ^c^	6.37 ± 1.16 ^c^	13.22 ± 1.17 ^d^
Phosphatidylinositols (% from total PI)						
C_20:5_ PI	36.16 ± 1.75 ^a^	28.21 ± 0.51 ^b^	35.57 ± 0.90 ^a^	6.41 ± 0.90 ^c^	9.20 ± 0.87 ^c^	13.87 ± 2.03 ^d^
C_20:4_ PI	40.25 ± 2.75 ^a^	51.30 ± 2.54 ^b^	31.93 ± 1.23 ^c^	86.49 ± 1.00 ^d^	82.23 ± 3.03 ^d^	76.21 ± 2.71 ^e^
odd-chain PI	31.34 ± 2.26 ^a^	37.36 ± 1.41 ^b^	24.39 ± 1.98 ^c^	54.36 ± 1.89 ^d^	56.24 ± 3.97 ^d^	47.98 ± 2.08 ^e^
very long chain PI	34.25 ± 2.07 ^a^	29.96 ± 1.22 ^a^	43.61 ± 3.08 ^b^	35.45 ± 0.60 ^a^	25.30 ± 2.42 ^c^	31.44 ± 1.71 ^a^
Phosphatidylserines (% from total PS)						
C_20:5_ PS	10.99 ± 1.11 ^a^	13.66 ± 1.45 ^b^	27.19 ± 0.69 ^c^	2.73 ± 0.28 ^d^	3.30 ± 0.45 ^d^	3.56 ± 0.90 ^d^
C_20:4_ PS	20.75 ± 2.09	23.60 ± 2.59 ^a^	16.93 ± 1.94 ^b^	22.33 ± 0.45	21.56 ± 3.94	18.90 ± 4.02
odd-chain PS	49.62 ± 1.86 ^a^	46.41 ± 1.04 ^b^	32.67 ± 0.99 ^c^	50.42 ± 0.94 ^a^	48.78 ± 1.01 ^b^	54.26 ± 1.62 ^d^
very long chain PS	77.04 ± 1.52 ^a^	75.42 ± 2.52 ^a^	79.59 ± 2.51 ^a^	83.27 ± 1.46 ^b^	80.75 ± 1.75 ^b^	87.32 ± 3.27 ^b^
C_22:6_ PS	1.02 ± 0.11	1.11 ± 0.26	6.40 ± 0.76 ^a^	0.41 ± 0.02	0.10 ± 0.06	0.67 ± 0.24 ^b^
Monounsaturated PS	37.88 ± 1.72 ^a^	34.95 ± 2.07 ^a^	20.22 ± 2.56 ^c^	49.61 ± 0.75 ^d^	49.99 ± 3.11 ^d^	51.88 ± 3.99 ^d^

Values within the same row bearing different superscripts differ (HSD test, *p* < 0.05). PE: glycerophosphoethanolamines; PC: glycerophosphocholines, PI: glycerophosphoinositols; PS: glycerophosphoserines.

## Data Availability

Not applicable.

## Supplementary Materials

The following supporting information can be downloaded at: www.mdpi.com/1660-3397/20/9/578/s1, Table S1: The content of molecular species of phospholipids in the different tissues (body wall, respiratory tree (RT) and intestine) of different origins (wild and cultured animals) of the sea cucumber *Apostichopus japonicus* and the results of two-factor ANOVA, Table S2: Pairwise comparison of the studied tissues of wild and cultivated sea cucumbers of the sea cucumber *Apostichopus japonicus* based on the results HSD test (*p* < 0.05).
