# Supplementary material for: The Phospholipid Molecular Species Profile of Apostichopus japonicus Tissues Modifies through Exposure to n-3 Polyunsaturated Fatty Acid-Deficient Diet
Source: Mar Drugs. 2022 Sep 15;20(9):578. doi: 10.3390/md20090578 (PMC9503100; doi:10.3390/md20090578)
Supplement: Supplementary file 1 [file marinedrugs-20-00578-s001.zip › Supplementary material_Table S1_Ermolenko.pdf]

Supplementary material

Article

The phospholipid molecular species profile of *Apostichopus japonicus* tissues modifies through exposure to n-3 polyunsaturated fatty acid-deficient diet

Ekaterina V. Ermolenko [1,*], Tatyana V. Sikorskaya [1] and Valeria P. Grigorchuk [2]

[1] A.V. Zhirmunsky National Scientific Center of Marine Biology, Far Eastern Branch, Russian Academy of Sciences, ul. Palchevskogo 17, 690041 Vladivostok, Russia;

[2] Federal Scientific Center of the East Asia Terrestrial Biodiversity, Far Eastern Branch, Russian Academy of Sciences, 159 Pr-t 100-let Vladivostoka Str., 690022 Vladivostok, Russia

* Correspondence: ecrire_711@mail.ru

Table S1 The content of molecular species of phospholipids in the different tissues (body wall, respiratory tree (RT) and intestine) of different origins (wild and cultured animals) of the sea cucumber *Apostichopus japonicus* and the results of two-factor ANOVA

Table S1. The content of molecular species of phospholipids in the different tissues (body wall, respiratory tree (RT) and intestine) of different origins (wild and cultured animals) of the sea cucumber *Apostichopus japonicus* and the results of two-factor ANOVA

Molecular species pf PL		Wild			Cultured			$F_{1,23}$, p	Factors		
		Body wall	RT	Intestine	Body wall	RT	Intestine		Origin	Tissue	Origin×Tissue
Phosphatidylethanolamines (% from total PE)											
1	18:1alk/18:1[1] PE	1.261±0.105	0.582±0.175	0.372±0.109	1.182±0.329	1.067±0.138	0.472±0.117	F	5.264	39.383	5.127
								p	0.034	0	0.017
2	37:6alk PE	0.427±0.106	0.603±0.228	0.499±0.051	0.177±0.015	0.222±0.07	0.224±0.084	F	42.231	1.887	0.759
								p	0	0.18	0.482
3	17:1alk/20:4 PE	0.446±0.104	0.559±0.204	0.204±0.035	0.774±0.343	0.824±0.198	0.447±0.198	F	11.203	7.106	0.094
								p	0.004	0.005	0.911
4	18:1alk/19:1 PE	1.507±0.405	1.155±0.156	0.313±0.027	2.099±0.308	1.93±0.521	0.457±0.099	F	16.182	48.398	2.243
								p	0.001	0	0.135
5	18:1alk/20:5 PE	22.149±1.672	20.701±1.625	30.177±1.04	6.964±0.433	5.162±1.224	15.352±2.182	F	640.261	102.883	0.118
								p	0	0	0.889
6	18:1alk/20:4 PE	22.123±0.766	20.097±1.648	11.57±0.712	29.702±0.562	29.899±1.663	22.365±2.361	F	254.817	93.287	2.612
								p	0	0	0.101
7	18:0alk/20:4 PE	2.523±0.763	0.96±0.172	0.779±0.267	3.461±0.627	1.248±0.216	0.993±0.109	F	7.3	56.726	1.682
								p	0.015	0	0.214
8	18:1alk/20:2 PE	1.768±0.318	1.374±0.346	1.191±0.098	1.072±0.216	1.238±0.291	0.761±0.215	F	15.572	6.262	2.31
								p	0.001	0.009	0.128
9	18:1alk/20:1; 19:1alk/19:1 PE	1.583±0.038	1.297±0.041	0.567±0.105	1.793±0.068	2.861±0.559	0.801±0.316	F	37.462	57.755	16.716
								p	0	0	0
10	19:1alk/20:5 PE	4.747±1.206	8.01±0.194	8.552±0.366	3.343±0.244	4.989±0.903	6.161±1.693	F	34.637	26.429	1.486
								p	0	0	0.253
11	18:1/20:5[2] PE	0.786±0.774	0.282±0.076	0.634±0.208	0±0	0±0	0±0	F	17.89	1.237	1.237
								p	0.001	0.314	0.314
12	19:1alk/20:4 PE	8.692±1.522	10.684±1.956	5.142±0.644	15.06±1.258	17.741±2.274	11.605±1.962	F	92.185	24.157	0.098
								p	0	0	0.907

13	18:0/20:5; 18:1/20:4 PE	2.286±0.947	2.869±1.169	5.717±1.527	1.018±0.392	0.732±0.459	1.935±0.324	F	40.804	13.98	3.862
								p	0	0	0.04
14	19:0alk/20:4 PE	0.705±0.501	1.135±0.223	0.392±0.172	1.459±0.214	1.841±0.589	0.867±0.382	F	17.248	10.193	0.307
								p	0.001	0.001	0.739
15	18:0/20:4 PE	1.913±0.495	2.693±0.092	2.115±0.354	2.15±0.17	0.948±0.467	2.237±0.259	F	11.105	2.217	21.456
								p	0.004	0.138	0
16	18:1alk/22:6 PE	4.348±0.196	3.051±0.811	8.295±0.59	2.762±0.16	2.463±0.284	5.576±1.242	F	35.537	87.66	5.063
								p	0	0	0.018
17	18:1alk/22:5; 18:0alk/22:6 PE	4.898±0.91	4.134±0.276	6.154±0.451	4.747±0.233	6.203±1.783	5.573±0.681	F	1.489	2.806	5.056
								p	0.238	0.087	0.018
18	20:1alk/20:4; 18:0alk/22:5 PE	3.193±0.228	3.484±0.408	2.656±0.29	5.396±0.418	6.822±1.018	5.242±0.922	F	111.753	7.806	1.692
								p	0	0.004	0.212
19	19:1/20:4 PE	0.833±0.321	1.5±0.261	1.905±0.193	1.038±0.092	0.562±0.067	1.355±0.38	F	17.985	18.59	11.074
								p	0	0	0.001
20	19:0/20:4 PE	0.707±0.144	1.421±0.087	0.553±0.105	1.971±0.087	1.049±0.33	2.045±0.288	F	95.115	0.556	51.971
								p	0	0.583	0
21	19:1alk/22:6 PE	0.77±0.164	0.926±0.07	1.868±0.182	0.753±0.305	1.018±0.029	2.226±0.48	F	1.925	58.617	1.153
								p	0.182	0	0.338
22	19:1alk/22:5 PE	1.183±0.229	1.63±0.136	1.413±0.117	2.281±0.112	3.556±0.556	2.67±0.53	F	106.609	13.28	3.374
								p	0	0	0.057
23	20:1/20:5 PE	2.047±0.473	1.846±0.426	2.634±0.484	0.35±0.167	0.12±0.021	0.889±0.317	F	139.002	10.103	0.009
								p	0	0.001	0.991
24	19:1alk/22:4; 21:1alk/20:4 PE	0.685±0.205	1.167±0.148	0.405±0.061	1.519±0.065	2.681±0.393	1.744±0.242	F	190.876	39.251	5.264
								p	0	0	0.016
25	20:1/20:4 PE	2.213±1.432	3.183±0.437	3.218±0.437	1.847±0.319	1.009±0.457	2.142±0.471	F	17.614	2.07	3.356
								p	0.001	0.155	0.058
26	20:0/20:4 PE	1.813±0.552	0.939±0.219	0.69±0.161	2.073±0.229	0.984±0.054	2.277±0.353	F	25.649	20.696	15
								p	0	0	0
27	20:1alk/22:5 PE	0.75±0.116	0.754±0.035	0.755±0.07	0.915±0.236	1.04±0.093	0.919±0.154	F	14.132	0.581	0.549
								p	0.001	0.569	0.587
28	21:0/20:5; 21:1/20:4 PE	0.873±0.04	0.833±0.027	0.348±0.074	0.822±0.083	0.357±0.071	0.635±0.189	F	4.091	28.924	31.576
								p	0.058	0	0

29	21:0/20:4 PE	0.469±0.069	0.51±0.148	0.145±0.026	1.406±0.124	0.703±0.189	0.861±0.085	F	158.736	28.798	20.422
								p	0	0	0
30	22:0/20:4 PE	0.452±0.053	0.431±0.135	0.217±0.01	0.696±0.046	0.288±0.101	0.381±0.114	F	6.092	21.6	10.783
								p	0.024	0	0.001
31	23:1/20:5 PE	0.893±0.049	0.554±0.076	0.303±0.024	0.361±0.04	0.14±0.052	0.241±0.074	F	222.555	91.881	39.164
								p	0	0	0
32	23:1/20:4 PE	0.958±0.185	0.636±0.035	0.219±0.016	0.809±0.109	0.304±0.073	0.544±0.335	F	0.588	20.909	8.394
								p	0.453	0	0.003
	$C_{20:5}$ PE	30.622±1.263	31.394±1.606	42.3±1.739	11.017±0.666	10.41±0.388	22.643±4.195	F	571.901	84.975	0.289
								p	0	0	0.753
	$C_{20:4}$ PE	43.847±1.536	44.749±3.218	27.149±0.93	62.447±0.544	57.4±2.783	48.121±4.004	F	289.711	90.41	5.855
								p	0	0	0.011
	$C_{22:6}$ PE	10.016±1.21	8.11±1.105	16.317±1.113	8.261±0.578	9.684±1.911	13.375±0.861	F	4.509	62.91	7.601
								p	0.048	0	0.004
	Alkyl/acyl PE	83.757±2.1	82.302±1.654	81.303±2.294	85.458±0.179	92.805±1.05	84.455±1.539	F	59.277	16.852	16.791
								p	0	0	0
	Diacyl PE	16.243±2.1	17.698±1.654	18.697±2.294	14.542±0.179	7.195±1.05	15.545±1.539	F	59.277	16.852	16.791
								p	0	0	0
Phosphatidylcholines (% from total PC)											
1	16:1alk/20:5; 16:2alk/20:4 PC	1.821±0.151	2.054±0.35	1.682±0.299	2.461±0.565	3.157±0.363	2.601±0.436	F	32.347	3.938	0.747
								p	0	0.038	0.488
2	16:0alk/20:5 PC	2.697±0.061	2.956±0.211	2.084±0.335	0±0	0±0	0±0	F	1492.725	14.998	14.998
								p	0	0	0
3	16:1alk/20:4 PC	0±0	0±0	0±0	5.052±0.957	5.046±0.325	3.776±0.598	F	558.513	4.706	4.706
								p	0	0.023	0.023
4	16:0alk/20:4 PC	0.577±0.22	0.894±0.285	1.103±0.086	2.908±0.552	1.748±0.492	1.243±0.33	F	55.792	5.283	18.912
								p	0	0.016	0
5	18:1alk/18:1 PC	1.643±0.341	1.001±0.295	0.244±0.023	0.202±0.025	0.308±0.11	0.157±0.044	F	90.303	29.276	25.269
								p	0	0	0
6	15:1alk/22:5 PC	0.647±0.023	0.756±0.148	0.519±0.052	1.003±0.155	0.656±0.221	0.818±0.131	F	10.65	2.782	6.442
								p	0.004	0.089	0.008
7	16:1/20:5 PC	2.565±0.209	2.632±0.491	1.49±0.225	0±0	0.222±0.116	0.267±0.077	F	432.569	10.932	18.196

								p	0	0.001	0
8	17:0alk/20:5 PC	1.698±0.169	1.871±0.366	1.361±0.029	0±0	0±0	0±0	F	595.892	4.953	4.953
								p	0	0.019	0.019
9	17:1alk/20:4 PC	0±0	0±0	0±0	2.99±0.663	3.283±0.181	1.957±0.265	F	499.539	10.734	10.734
								p	0	0.001	0.001
10	16:0/20:5 PC	5.2±1.433	3.037±1.102	3.891±0.973	0.436±0.115	0.666±0.161	1.571±0.068	F	83.991	3.225	5.499
								p	0	0.064	0.014
11	37:4alk PC	0.612±0.216	0.813±0.182	0.276±0.128	3.743±0.322	2.929±1.001	1.262±0.154	F	126.686	21.461	11.266
								p	0	0	0.001
12	36:3 PC	1.332±0.243	2.409±0.798	1.503±0.654	0.371±0.096	0.833±0.359	0.527±0.196	F	37.962	6.067	1.135
								p	0	0.01	0.343
13	18:1/18:1 PC	1.799±0.13	1.598±0.395	1.321±0.145	0.115±0.014	0.282±0.055	0.785±0.177	F	219.041	0.779	18.066
								p	0	0.474	0
14	38:7alk PC	0.796±0.022	0.859±0.126	0.707±0.084	1.231±0.076	1.051±0.102	1.498±0.4	F	40.237	1.325	5.463
								p	0	0.291	0.014
15	18:1alk/20:5; 18:2alk/20:4 PC	11.138±0.715	13.242±1.39	11.464±0.355	11.892±0.574	13.499±0.627	12.661±2.755	F	1.795	4.008	0.244
								p	0.197	0.036	0.786
16	18:0alk/20:5 PC	9.649±0.715	8.522±1.154	10.509±0.586	0±0	0±0	0±0	F	1505.247	5.451	5.451
								p	0	0.014	0.014
17	18:1alk/20:4 PC	0±0	0±0	0±0	20.431±0.839	19.415±1.66	12.697±0.538	F	2946.238	56.555	56.555
								p	0	0	0
18	37:5 PC	0.882±0.155	0.452±0.05	0.495±0.095	1.019±0.243	1.132±0.342	1.509±0.054	F	62.509	2.679	10.996
								p	0	0.096	0.001
19	18:0alk/20:4 PC	1.723±0.207	1.45±0.612	0.9±0.074	6.36±0.536	4.224±0.662	3.351±0.627	F	252.504	29.211	10.849
								p	0	0	0.001
20	38:3alk PC	0.574±0.345	0.481±0.129	0±0	0.579±0.33	0±0	0±0	F	3.687	16.44	3.821
								p	0.071	0	0.041
21	16:1alk/22:1 PC	0.254±0.079	0.148±0.155	0±0	0±0	0±0	0±0	F	21.422	6.47	6.47
								p	0	0.008	0.008
22	38:7 PC	2.097±0.107	1.899±0.301	1.788±0.232	0.179±0.029	0.398±0.225	0.719±0.09	F	374.154	0.913	10.035
								p	0	0.419	0.001
23	39:6alk PC	0.976±0.074	1.077±0.093	0.759±0.048	1.108±0.124	1.207±0.114	1.436±0.083	F	68.489	2.341	23.188

								p	0	0.125	0
24	18:1/20:5 PC	15.626±0.859	16.761±0.565	19.911±1.352	5.304±0.683	8.823±1.608	12.323±1.386	F	340.102	49.287	3.38
								p	0	0	0.057
25	19:0alk/20:5 PC	1.752±0.301	2.125±0.179	2.07±0.091	3.021±0.465	3.52±0.28	2.446±0.307	F	71.164	8.086	7.135
								p	0	0.003	0.005
26	18:0/20:5 PC	7.63±1.058	6.46±0.522	5.575±0.535	0±0	0±0	0±0	F	921.494	7.592	7.592
								p	0	0.004	0.004
27	18:1/20:4 PC	0±0	0±0	0±0	7.638±0.5	7.708±0.87	7.15±0.552	F	1544.221	0.847	0.847
								p	0	0.445	0.445
28	39:4alk PC	0.574±0.335	0.603±0.125	0.219±0.067	2.996±0.141	2.283±0.839	1.423±0.627	F	90.099	9.176	3.619
								p	0	0.002	0.048
29	18:0/20:4 PC	0.566±0.082	0.468±0.036	0.558±0.047	1.968±0.586	0.887±0.085	1.427±0.104	F	77.96	11.243	7.836
								p	0	0.001	0.004
30	38:3 PC	0.578±0.301	2.795±1.582	0.672±0.123	1.461±0.645	1.913±0.334	0.826±0.251	F	0.03	11.086	2.951
								p	0.865	0.001	0.078
31	38:7alk PC	1.339±0.157	1.405±0.321	1.247±0.052	1.08±0.451	0.476±0.057	2.136±0.289	F	0.856	16.53	24.131
								p	0.367	0	0
32	18:0alk/22:6 PC	2.344±0.265	2.644±0.375	4.164±0.3	1.796±0.313	1.741±0.366	3.577±0.223	F	28.522	83.456	0.783
								p	0	0	0.472
33	36:6 PC	1.177±0.193	1.353±0.241	1.346±0.112	0.825±0.09	1.393±0.201	1.99±0.29	F	1.84	22.298	12.584
								p	0.192	0	0
34	40:5alk PC	1.065±0.233	0.773±0.031	0.685±0.061	1.686±0.186	1.686±0.284	1.567±0.237	F	101.351	3.27	1.34
								p	0	0.061	0.287
35	39:5 PC	0.841±0.245	1.102±0.123	1.142±0.099	2.229±0.256	1.973±0.11	1.815±0.188	F	173.528	0.271	8.273
								p	0	0.765	0.003
36	40:4alk PC	0.234±0.024	0.088±0.019	0±0	0.224±0.107	0.238±0.088	0.198±0.165	F	9.648	4.284	3.017
								p	0.006	0.03	0.074
37	41:11alk PC	0.332±0.04	0.38±0.043	1.369±0.187	0±0	0±0	0±0	F	452.087	107.259	107.259
								p	0	0	0
38	40:10 PC	0.843±0.098	0.954±0.172	1.381±0.266	0.041±0.007	0.125±0.014	0.513±0.26	F	140.812	19.414	0.074
								p	0	0	0.929
39	40:9 PC	0.357±0.082	0.655±0.066	0.658±0.238	0.419±0.178	0.664±0.175	1.092±0.092	F	7.336	20.562	4.647

								p	0.014	0	0.024
40	18:1/22:6 PC	5.342±0.281	4.11±0.63	5.501±0.4	2.117±0.278	1.752±0.42	5.264±1.228	F	56.526	31.309	11.839
								p	0	0	0.001
41	20:1/20:5; 18:0/22:6 PC	4.799±0.212	4.86±0.556	6.12±0.212	3.233±0.237	2.881±0.634	4.378±0.416	F	108.567	26.71	0.5
								p	0	0	0.615
42	40:5 PC	1.272±0.122	1.393±0.289	1.207±0.285	1.415±0.148	1.284±0.165	1.892±0.669	F	3.054	1.023	2.913
								p	0.098	0.38	0.08
43	42:7alk PC	0.174±0.023	0.277±0.071	0.264±0.077	0±0	0.189±0.057	0.314±0.046	F	10.749	31.01	9.113
								p	0.004	0	0.002
44	42:11 PC	0.373±0.158	0.223±0.03	0.763±0.058	0±0	0±0	0.342±0.036	F	135.951	87.714	4.207
								p	0	0	0.032
45	42:7 PC	1.191±0.066	0.887±0.034	1.106±0.065	0±0	0.162±0.075	0±0	F	2377.603	3.986	48.21
								p	0	0.037	0
46	42:6 PC	1.536±0.134	0.589±0.009	0.544±0.169	0±0	0±0	0±0	F	611.233	80.838	80.838
								p	0	0	0
47	43:6 PC	0.656±0.034	0.664±0.08	0.491±0.065	0.469±0.122	0.245±0.051	0.491±0.111	F	35.077	3.456	12.657
								p	0	0.054	0
48	44:6 PC	0.716±0.13	0.28±0.076	0.911±0.204	0±0	0±0	0±0	F	226.026	19.497	19.497
								p	0	0	0
	$C_{20:5}$ PC	46.817±0.898	44.364±0.819	46.89±1.009	8.76±0.972	13.231±1.805	16.607±1.15	F	4934.627	25.342	27.197
								p	0	0	0
	$C_{20:4}$ PC	2.865±0.186	2.812±0.448	2.562±0.203	47.348±0.882	42.311±1.314	31.601±2.108	F	7071.473	111.726	103.18
								p	0	0	0
	Alkyl/acyl PC	42.618±2.679	44.418±3.327	41.626±1.241	70.762±0.942	66.656±1.915	55.118±0.767	F	654.544	39.099	26.156
								p	0	0	0
	Diacyl PC	57.382±2.679	55.582±3.327	58.374±1.241	29.238±0.942	33.344±1.915	44.882±0.767	F	654.544	39.099	26.156
								p	0	0	0
	$C_{22:6}$ PC	12.49±0.41	11.61±1.46	15.78±0.42	7.15±0.6	6.37±1.16	13.22±1.17	F	124.365	76.242	5.344
								p	0	0	0.015
Phosphatidylinositols (% from total PI)											
1	18:0alk/20:5 PI	0.695±0.193	0.562±0.18	0.923±0.212	0.237±0.095	0±0	0.878±0.236	F	25.285	27.051	4.996
								p	0	0	0.019

2	18:0alk/20:4 PI	0.174±0.037	0.249±0.078	0.201±0.042	0.364±0.171	0.193±0.039	0.675±0.083	F	31.5	13.332	18.051
								p	0	0	0
3	18:1/20:5 PI	0.905±0.275	0.583±0.103	1.058±0.178	0.071±0.056	0±0	0.173±0.126	F	154.738	9.342	2.28
								p	0	0.002	0.131
4	18:0/20:5 PI	5.084±1.402	3.052±0.633	4.161±0.421	0±0	0±0	0±0	F	237.819	4.882	4.882
								p	0	0.02	0.02
5	18:1/20:4 PI	0±0	0±0	0±0	1.112±0.286	0.822±0.584	1.482±0.276	F	93.473	2.629	2.629
								p	0	0.1	0.1
6	18:0/20:4 PI	1.68±0.167	1.961±0.187	1.463±0.167	2.228±0.657	1.523±0.313	1.79±0.398	F	0.98	1.699	4.136
								p	0.335	0.211	0.033
7	19:1/20:5 PI	0.229±0.086	0.186±0.076	0.149±0.044	0.054±0.039	0±0	0.094±0.058	F	34.715	1.411	3.156
								p	0	0.27	0.067
8	19:0/20:5 PI	2.178±0.595	2.072±0.281	2.094±0.217	1.301±0.447	2.14±0.269	2.162±0.179	F	2.801	2.924	4.549
								p	0.111	0.08	0.025
9	19:0/20:4 PI	1.9±0.309	3.368±0.735	1.488±0.164	6.52±0.243	8.786±2.785	5.133±1.62	F	67.463	8.612	0.854
								p	0	0.002	0.442
10	20:1/20:5 PI	7.223±0.961	3.674±0.475	5.705±1.521	0.75±0.207	0.979±0.891	1.386±0.212	F	167.451	8.164	9.924
								p	0	0.003	0.001
11	20:1/20:4; 20:0/20:5 PI	12.861±0.922	11.373±0.535	10.729±1.748	6.122±0.59	7.455±2.195	7.23±0.865	F	79.314	0.362	3.687
								p	0	0.701	0.046
12	20:0/20:4 PI	5.731±0.901	7.752±1.255	3.628±0.162	11.26±0.983	11.784±1.706	10.277±0.934	F	146.743	13.319	2.888
								p	0	0	0.082
13	20:1/20:2 PI	0.566±0.191	1.202±0.118	1.242±0.151	0±0	0±0	0±0	F	495.01	23.579	23.579
								p	0	0	0
14	21:1/20:5 PI	1.287±0.111	1.213±0.077	1.239±0.13	0.294±0.092	1.022±0.358	0.83±0.08	F	57.025	7.788	11.569
								p	0	0.004	0.001
15	21:0/20:5 PI	3.561±0.395	4.683±0.373	2.321±0.404	0±0	0±0	0±0	F	972.804	36.51	36.51
								p	0	0	0
16	21:1/20:4 PI	0±0	0±0	0±0	4.181±0.331	7.878±1.905	5.551±0.462	F	313.742	10.602	10.602
								p	0	0.001	0.001
17	21:0/20:4 PI	2.35±0.348	4.11±0.811	1.227±0.113	9.885±0.41	13.409±1.755	8.074±1.099	F	427.389	39.654	3.658
								p	0	0	0.046

18	22:2/20:5 PI	0.684±0.073	0.356±0.082	0.586±0.122	0±0	0±0	0.075±0.113	F	241.934	10.134	8.148
								p	0	0.001	0.003
19	22:1/20:5 PI	7.561±0.533	6.588±0.525	7.07±0.667	1.465±0.404	1.612±0.752	3.636±0.824	F	348.891	8.117	8.887
								p	0	0.003	0.002
20	22:1/20:4 PI	9.315±1.025	13.723±1.331	8.708±0.888	15.487±1.082	14.863±4.601	14.545±1.805	F	23.669	3.092	3.252
								p	0	0.07	0.062
21	22:0/20:4 PI	0.967±0.089	1.746±0.352	0.882±0.129	3.223±0.441	2.23±1.283	4.055±1.195	F	40.911	0.895	6.558
								p	0	0.426	0.007
22	22:1/20:2 PI	0.797±0.432	1.585±0.811	1.518±0.185	0±0	0±0	0.511±0.633	F	35.902	3.649	1.562
								p	0	0.047	0.237
23	23:1/20:5 PI	6.302±0.874	6.338±0.598	7.131±1.284	2.606±0.17	4.151±1.273	4.872±0.785	F	52.654	5.707	1.728
								p	0	0.012	0.206
24	23:1/20:4 PI	11.878±1.567	13.048±0.748	6.391±0.75	28.648±1.62	18.174±3.011	19.73±0.806	F	311.995	40.214	26.987
								p	0	0	0
25	23:0/20:4 PI	0.304±0.033	0.546±0.073	0.321±0.039	0.702±0.212	0.684±0.569	1.073±0.439	F	11.625	0.801	2.006
								p	0.003	0.464	0.163
26	23:1/20:2 PI	1.348±0.644	1.791±0.673	2.029±0.648	0.172±0.064	0±0	0.463±0.14	F	62.672	2.3	0.886
								p	0	0.129	0.429
27	24:3/20:5 PI	0.602±0.282	0.133±0.071	0.421±0.049	0±0	0±0	0±0	F	61.633	7.718	7.718
								p	0	0.004	0.004
28	24:1/20:5 PI	7.073±0.462	2.446±0.387	8.413±0.523	0.377±0.053	0.27±0.334	1.148±0.709	F	831.073	117.614	74.416
								p	0	0	0
29	24:1/20:4 PI	5.954±0.499	4.8±0.724	7.617±1.882	2.884±1.16	1.887±1.606	3.821±0.55	F	44.746	7.95	0.311
								p	0	0.003	0.737
30	24:0/20:4; 24:1/20:3 PI	0.33±0.12	0.414±0.085	0.69±0.118	0.057±0.05	0.138±0.171	0.337±0.028	F	47.94	19.454	0.36
								p	0	0	0.702
31	24:1/20:2 PI	0.464±0.35	0.444±0.09	10.595±3.601	0±0	0±0	0±0	F	40.409	31.409	31.409
								p	0	0	0
	$C_{20:5}$ PI	36.159±1.747	28.215±0.51	35.566±0.903	6.406±0.898	9.195±0.874	13.868±2.028	F	2024.436	44.5	38.175
								p	0	0	0
	$C_{20:4}$ PI	40.252±2.746	51.302±2.543	31.927±1.226	86.494±1.004	82.232±3.025	76.205±2.712	F	1786.784	62.86	25.229
								p	0	0	0

	odd-chain PI	31.338±2.259	37.356±1.413	24.39±1.981	54.363±1.895	56.245±3.971	47.982±2.075	F	494.824	39.827	2.28
								p	0	0	0.131
	long-chain PI	34.254±2.072	29.961±1.22	43.607±3.084	35.446±0.6	25.305±2.423	31.445±1.709	F	39.963	51.427	22
								p	0	0	0
Phosphatidylserines (% from total PS)											
1	18:0/20:5 PS	0.794±0.084	1.491±0.474	1.449±0.232	0.068±0.015	0.1±0.065	0.078±0.03	F	167.618	6.732	5.903
								p	0	0.007	0.011
2	18:0/20:4 PS	0.483±0.206	0.759±0.309	0.443±0.085	0.24±0.121	0.238±0.072	0.083±0.024	F	30.567	4.045	1.407
								p	0	0.035	0.271
3	18:1/20:1 PS	0.823±0.182	0.892±0.11	1.082±0.231	0.152±0.017	0.077±0.032	0.095±0.092	F	226.005	1.55	2.785
								p	0	0.239	0.088
4	18:0/20:1 PS	0.832±0.153	0.985±0.1	0.854±0.382	0.649±0.109	0.741±0.269	0.347±0.116	F	12.606	2.981	1.283
								p	0.002	0.076	0.301
5	19:0/20:4 PS	0.433±0.121	0.696±0.207	0.273±0.082	0.701±0.216	0.744±0.352	0.263±0.105	F	1.525	10.326	1.055
								p	0.233	0.001	0.369
6	20:1/20:5 PS	1.132±0.159	1.033±0.178	1.661±0.087	0.071±0.045	0.077±0.018	0.024±0.029	F	790.513	16.925	23.898
								p	0	0	0
7	20:0/20:5; 20:1/20:4 PS	2.604±0.278	2.732±0.426	4.553±0.592	0.891±0.128	1.043±0.128	0.541±0.147	F	331.277	13.188	32.194
								p	0	0	0
8	20:0/20:4 PS	2.71±0.408	2.634±0.501	1.6±0.366	2.625±0.224	2.122±0.143	1.356±0.302	F	3.97	25.899	0.785
								p	0.062	0	0.471
9	22:1/18:1; 20:1/20:1 PS	3.354±0.377	2.977±0.158	2.331±0.5	2.377±0.072	2.454±0.436	1.447±0.236	F	33.995	19.867	1.033
								p	0	0	0.376
10	21:0/20:5 PS	1.957±0.113	2.433±0.285	2.59±0.275	1.028±0.217	1.319±0.11	0.905±0.174	F	214.732	7.043	7.206
								p	0	0.006	0.005
11	21:0/20:4 PS	2.841±0.565	3.549±0.572	1.231±0.221	3.183±0.463	4.38±1.402	3.425±1.54	F	8.645	6.174	2.107
								p	0.009	0.009	0.151
12	23:1/18:1; 22:1/19:1 PS	5±0.503	4.399±0.354	2.339±0.426	4.749±0.296	5.96±0.532	4.113±1.32	F	14.227	19.818	5.559
								p	0.001	0	0.013
13	22:1/20:5 PS	2.326±0.501	2.63±0.366	5.028±0.206	0.768±0.078	0.912±0.083	0.736±0.095	F	509.431	54.565	62.822
								p	0	0	0
14	22:1/20:4 PS	4.655±0.543	5.841±0.203	6.531±0.72	4.386±0.092	4.677±1.177	3.777±0.318	F	29.852	3.263	8.087

								p	0	0.062	0.003
15	22:0/20:4 PS	1.955±0.223	2.231±0.38	1.374±0.288	2.919±0.691	2.945±0.241	3.099±0.491	F	43.958	1.416	3.155
								p	0	0.268	0.067
16	42:3 PS	0.934±0.061	1.493±0.622	1.115±0.365	1.511±0.193	2.482±0.471	1.388±0.273	F	15.817	10.534	1.81
								p	0.001	0.001	0.192
17	22:1/20:1; 24:1/18:1 PS	10.725±0.511	10.505±1.332	5.874±0.682	15.338±0.379	17.141±1.254	14.016±1.173	F	268.829	36.023	6.727
								p	0	0	0.007
18	23:1/20:5 PS	3.1±0.37	3.831±0.454	5.595±0.207	0.794±0.061	0.898±0.429	1.818±0.806	F	265.764	33.163	5.338
								p	0	0	0.015
19	23:1/20:4 PS	7.675±0.829	7.889±0.868	5.478±0.75	8.28±0.429	6.458±1.248	6.956±1.393	F	0.299	6.566	4.704
								p	0.591	0.007	0.023
20	23:1/20:2 PS	2.056±0.201	1.922±0.333	1.047±0.428	2.04±0.286	1.805±1.071	2.764±0.901	F	4.227	0.189	5.368
								p	0.055	0.829	0.015
21	23:1/20:1; 22:1/21:1 PS	18.854±0.866	15.359±0.888	6.811±0.894	23.363±0.795	22.167±1.388	26.565±3.147	F	260.886	15.865	54.76
								p	0	0	0
22	24:1/20:5 PS	1.683±0.402	2.243±0.279	10.864±1.117	0±0	0±0	0±0	F	587.913	213.556	213.556
								p	0	0	0
23	22:1/22:5 PS	0±0	0±0	0±0	0.869±0.072	0.631±0.232	1.284±0.078	F	475.178	20.09	20.09
								p	0	0	0
24	24:1/20:4; 22:1/22:4 PS	3.669±0.628	3.212±0.236	8.362±1.165	3.063±0.178	2.291±0.675	3.227±1.194	F	47.736	33.401	20.624
								p	0	0	0
25	22:1/22:2 PS	1.703±0.214	1.522±0.522	1.108±0.467	1.357±0.117	1.243±0.633	1.644±0.413	F	0.029	0.324	2.574
								p	0.868	0.727	0.104
26	24:1/20:1; 23:1/21:1 PS	8.304±0.45	9.087±0.188	7.54±1.536	10.912±0.337	10.684±0.67	11.296±1.537	F	45.91	0.48	2.537
								p	0	0.626	0.107
27	23:1/22:6 PS	0.9±0.059	0.911±0.185	2.778±0.246	0.406±0.021	0.102±0.059	0.668±0.238	F	293.698	133.395	55.532
								p	0	0	0
28	23:1/22:5 PS	0.586±0.212	0.794±0.253	1.764±0.165	0.549±0.183	0.35±0.087	0.82±0.498	F	19.086	19.625	5.834
								p	0	0	0.011
29	23:1/22:4 PS	2.507±0.617	1.124±0.115	0.649±0.386	1.704±0.379	1.312±0.368	2.58±0.705	F	5.248	7.194	17.438
								p	0.034	0.005	0
30	23:1/22:2 PS	1.529±0.239	1.418±0.371	0.751±0.148	1.297±0.288	1.003±0.39	1.582±0.312	F	0.245	1.507	9.886

								p	0.627	0.248	0.001
31	24:1/21:1; 23:1/22:1 PS	2.182±0.082	2.084±0.236	1.361±0.312	2.328±0.344	2.285±1.133	1.799±0.538	F	1.325	3.564	0.156
								p	0.265	0.05	0.857
32	24:1/22:6 PS	0.125±0.101	0.203±0.078	3.624±0.601	0±0	0±0	0±0	F	165.621	126.919	126.919
								p	0	0	0
33	24:1/22:4 PS	1.14±0.098	0.735±0.144	1.266±0.454	1.192±0.22	1.221±0.178	1.125±0.061	F	1.966	2.089	3.858
								p	0.178	0.153	0.04
34	24:1/22:2 PS	0.426±0.057	0.387±0.151	0.67±0.113	0.189±0.052	0.141±0.019	0.178±0.018	F	90	7.786	5.99
								p	0	0.004	0.01
	$C_{20:5}$ PS	10.994±1.113	13.66±1.453	27.188±0.686	2.729±0.277	3.305±0.446	3.561±0.901	F	1454.802	199.868	169.73
								p	0	0	0
	$C_{20:4}$ PS	20.752±2.094	23.598±2.592	16.932±1.941	22.334±0.448	21.564±3.935	18.96±4.021	F	0.212	6.079	1.275
								p	0.651	0.01	0.304
	odd-chain PS	49.62±1.861	46.408±1.038	32.668±0.99	50.422±0.941	48.782±1.012	54.258±1.619	F	244.023	52.495	159.915
								p	0	0	0
	very long chain PS	77.036±1.52	75.422±2.521	79.592±2.507	83.266±1.457	80.747±1.751	87.322±3.272	F	48.225	11.427	0.574
								p	0	0.001	0.573
	$C_{22:6}$ PS	1.02±0.11	1.11±0.26	6.4±0.76	0.41±0.02	0.1±0.06	0.67±0.24	F	341.203	20.417	34.179
								p	0	0	0
	Monounsaturated PS	37.88±1.72	34.95±2.07	20.22±2.56	49.61±0.75	49.99±3.11	51.88±3.99	F	4.509	62.91	7.601
								p	0.048	0	0.004

Data were presented as mean ± SD, $n = 4$

[1]*sn*-1 alkyl/ *sn*-2 acyl

[2]*sn*-1(2) acyl/ *sn*-2(1) acyl
