# Supplementary material for: The Phospholipid Molecular Species Profile of Apostichopus japonicus Tissues Modifies through Exposure to n-3 Polyunsaturated Fatty Acid-Deficient Diet
Source: Mar Drugs. 2022 Sep 15;20(9):578. doi: 10.3390/md20090578 (PMC9503100; doi:10.3390/md20090578)
Supplement: Supplementary file 1 [file marinedrugs-20-00578-s001.zip › Supplementary material_Table S2_Ermolenko.pdf]

Supplementary material

*Article*

# The phospholipid molecular species profile of *Apostichopus japonicus* tissues modifies through exposure to n-3 polyunsaturated fatty acid-deficient diet

**Ekaterina V. Ermolenko [1,*], Tatyana V. Sikorskaya [1] and Valeria P. Grigorchuk [2]**

[1] A.V. Zhirmunsky National Scientific Center of Marine Biology, Far Eastern Branch,
Russian Academy of Sciences, ul. Palchevskogo 17, 690041 Vladivostok, Russia

[2] Federal Scientific Center of the East Asia Terrestrial Biodiversity, Far Eastern Branch, Russian Academy of Sciences, 159 Pr-t 100-let Vladivostoka Str., 690022
Vladivostok, Russia

* Correspondence: ecrire_711@mail.ru

**Table S2**. Pairwise comparison of the studied tissues of wild and cultivated sea cucumbers of the sea cucumber *Apostichopus japonicus* based on the results HSD test ($p < 0.05$)

| Group | Molecular species pf PL | P value | Group | Molecular species pf PL | P value | Group | Molecular species pf PL | P value |
|---|---|---|---|---|---|---|---|---|
| Body wall | | | Respiratory tree | | 0.0048 | Intestine | | 0.0014 |
| Wild vs cultured | 37:6alk PE | 0.0034 | Wild vs cultured | 18:1alk/18:1[1] PE | | Wild vs cultured | 37:6alk PE | |
| | 18:1alk/20:5 PE | 0.0000 | | 37:6alk PE | 0.0184 | | 18:1alk/19:1 PE | 0.0311 |
| | 18:1alk/20:4 PE | 0.0000 | | 18:1alk/19:1 PE | 0.0290 | | 18:1alk/20:5 PE | 0.0000 |
| | 18:1alk/20:2 PE | 0.0111 | | 18:1alk/20:5 PE | 0.0000 | | 18:1alk/20:4 PE | 0.0001 |
| | 18:1alk/20:1; 19:1alk/19:1 PE | 0.0017 | | 18:1alk/20:4 PE | 0.0002 | | 18:1alk/20:2 PE | 0.0109 |
| | 19:1alk/20:4 PE | 0.0007 | | 18:1alk/20:1; 19:1alk/19:1 PE | 0.0014 | | 19:1alk/20:5 PE | 0.0328 |
| | 18:0/20:5; 18:1/20:4 PE | 0.0481 | | 19:1alk/20:5 PE | 0.0006 | | 18:1/20:5 PE | 0.0009 |
| | 19:0alk/20:4 PE | 0.0324 | | 18:1/20:5[2] PE | 0.0003 | | 19:1alk/20:4 PE | 0.0008 |
| | 18:1alk/22:6 PE | 0.0000 | | 19:1alk/20:4 PE | 0.0033 | | 18:0/20:5; 18:1/20:4 PE | 0.0029 |

| | | | | | |
|---|---|---|---|---|---|
| 20:1alk/20:4; 18:0alk/22:5 PE | 0.0000 | 18:0/20:5; 18:1/20:4 PE | 0.0144 | 18:1alk/22:6 PE | 0.0075 |
| 19:0/20:4 PE | 0.0000 | 18:0/20:4 PE | 0.0003 | 20:1alk/20:4; 18:0alk/22:5 PE | 0.0017 |
| 19:1alk/22:5 PE | 0.0001 | 20:1alk/20:4; 18:0alk/22:5 PE | 0.0009 | 19:1/20:4 PE | 0.0417 |
| 20:1/20:5 PE | 0.0005 | 19:1/20:4 PE | 0.0004 | 19:0/20:4 PE | 0.0000 |
| 19:1alk/22:4; 21:1alk/20:4 PE | 0.0002 | 19:1alk/22:5 PE | 0.0005 | 19:1alk/22:5 PE | 0.0036 |
| 21:0/20:4 PE | 0.0000 | 20:1/20:5 PE | 0.0002 | 20:1/20:5 PE | 0.0009 |
| 22:0/20:4 PE | 0.0004 | 19:1alk/22:4; 21:1alk/20:4 PE | 0.0003 | 19:1alk/22:4; 21:1alk/20:4 PE | 0.0000 |
| 23:1/20:5 PE | 0.0003 | 20:1/20:4 PE | 0.0005 | 20:1/20:4 PE | 0.0155 |
| 16:0alk/20:5 PC | 0.0000 | 20:1alk/22:5 PE | 0.0012 | 20:0/20:4 PE | 0.0002 |
| 16:1alk/20:4 PC | 0.0000 | 21:0/20:5; 21:1/20:4 PE | 0.0000 | 21:0/20:5; 21:1/20:4 PE | 0.0299 |
| 16:0alk/20:4 PC | 0.0002 | 23:1/20:5 PE | 0.0001 | 21:0/20:4 PE | 0.0000 |
| 18:1alk/18:1 PC | 0.0002 | 23:1/20:4 PE | 0.0002 | 22:0/20:4 PE | 0.0283 |
| 15:1alk/22:5 PC | 0.0039 | 16:1alk/20:5; 16:2alk/20:4 PC | 0.0147 | 16:1alk/20:5; 16:2alk/20:4 PC | 0.0132 |
| 16:1/20:5 PC | 0.0000 | 16:0alk/20:5 PC | 0.0000 | 16:0alk/20:5 PC | 0.0000 |
| 17:0alk/20:5 PC | 0.0000 | 16:1alk/20:4 PC | 0.0000 | 16:1alk/20:4 PC | 0.0000 |
| 17:1alk/20:4 PC | 0.0001 | 18:1alk/18:1 PC | 0.0047 | 18:1alk/18:1 PC | 0.0131 |
| 16:0/20:5 PC | 0.0006 | 15:1alk/22:5 PC | 0.0054 | 15:1alk/22:5 PC | 0.0055 |
| 37:4alk PC | 0.0000 | 16:1/20:5 PC | 0.0001 | 16:1/20:5 PC | 0.0000 |
| 36:3 PC | 0.0003 | 17:0alk/20:5 PC | 0.0004 | 17:0alk/20:5 PC | 0.0000 |
| 18:1/18:1 PC | 0.0000 | 17:1alk/20:4 PC | 0.0000 | 17:1alk/20:4 PC | 0.0000 |
| 38:7alk PC | 0.0000 | 16:0/20:5 PC | 0.0048 | 16:0/20:5 PC | 0.0031 |
| 18:0alk/20:5 PC | 0.0000 | 36:3 PC | 0.0031 | 37:4alk PC | 0.0000 |
| 18:1alk/20:4 PC | 0.0000 | 18:1/18:1 PC | 0.0014 | 36:3 PC | 0.0289 |
| 18:0alk/20:4 PC | 0.0000 | 38:7alk PC | 0.0027 | 18:1/18:1 PC | 0.0034 |
| 16:1alk/22:1 PC | 0.0007 | 18:1alk/20:5; 18:2alk/20:4 PC | 0.0002 | 38:7alk PC | 0.0083 |
| 38:7 PC | 0.0000 | 18:0alk/20:5 PC | 0.0000 | 18:0alk/20:5 PC | 0.0000 |
| 18:1/20:5 PC | 0.0000 | 18:1alk/20:4 PC | 0.0000 | 18:1alk/20:4 PC | 0.0000 |
| 19:0alk/20:5 PC | 0.0038 | 38:3alk PC | 0.0000 | 37:5 PC | 0.0000 |
| 18:0/20:5 PC | 0.0000 | 39:6alk PC | 0.0000 | 18:0alk/20:4 PC | 0.0002 |
| 18:1/20:4 PC | 0.0000 | 18:1/20:5 PC | 0.0000 | 38:7 PC | 0.0001 |
| 39:4alk PC | 0.0000 | 19:0alk/20:5 PC | 0.0035 | 39:6alk PC | 0.0000 |
| 18:0/20:4 PC | 0.0032 | 18:0/20:5 PC | 0.0000 | 18:1/20:5 PC | 0.0002 |

| | | | | | |
|---|---|---|---|---|---|
| 38:3 PC | 0.0478 | 18:1/20:4 PC | 0.0000 | 18:0/20:5 PC | 0.0000 |
| 18:0alk/22:6 PC | 0.0369 | 18:0/20:4 PC | 0.0000 | 18:1/20:4 PC | 0.0000 |
| 36:6 PC | 0.0164 | 38:3 PC | 0.0375 | 39:4alk PC | 0.0088 |
| 40:5alk PC | 0.0059 | 38:7alk PC | 0.0004 | 18:0/20:4 PC | 0.0000 |
| 39:5 PC | 0.0002 | 18:0alk/22:6 PC | 0.0006 | 38:7alk PC | 0.0009 |
| 41:11alk PC | 0.0000 | 36:6 PC | 0.0005 | 18:0alk/22:6 PC | 0.0200 |
| 40:10 PC | 0.0000 | 39:5 PC | 0.0005 | 36:6 PC | 0.0060 |
| 18:1/22:6 PC | 0.0000 | 41:11alk PC | 0.0000 | 40:5alk PC | 0.0004 |
| 20:1/20:5; 18:0/22:6 PC | 0.0001 | 40:10 PC | 0.0001 | 39:5 PC | 0.0007 |
| 42:7alk PC | 0.0000 | 40:9 PC | 0.0002 | 41:11alk PC | 0.0000 |
| 42:11 PC | 0.0032 | 18:1/22:6 PC | 0.0001 | 40:10 PC | 0.0034 |
| 42:7 PC | 0.0000 | 20:1/20:5; 18:0/22:6 PC | 0.0000 | 40:9 PC | 0.0146 |
| 42:6 PC | 0.0000 | 40:5 PC | 0.0027 | 20:1/20:5; 18:0/22:6 PC | 0.0003 |
| 43:6 PC | 0.0260 | 42:7alk PC | 0.0031 | 42:11 PC | 0.0000 |
| 44:6 PC | 0.0000 | 42:11 PC | 0.0000 | 42:7 PC | 0.0000 |
| 18:0alk/20:5 PI | 0.0054 | 42:7 PC | 0.0000 | 42:6 PC | 0.0007 |
| 18:1/20:5 PI | 0.0010 | 42:6 PC | 0.0000 | 44:6 PC | 0.0001 |
| 18:0/20:5 PI | 0.0003 | 43:6 PC | 0.0000 | 18:0alk/20:4 PI | 0.0000 |
| 18:1/20:4 PI | 0.0002 | 44:6 PC | 0.0009 | 18:1/20:5 PI | 0.0002 |
| 19:1/20:5 PI | 0.0098 | 18:0alk/20:5 PI | 0.0008 | 18:0/20:5 PI | 0.0000 |
| 19:0/20:4 PI | 0.0000 | 18:1/20:5 PI | 0.0000 | 18:1/20:4 PI | 0.0000 |
| 20:1/20:5 PI | 0.0001 | 18:0/20:5 PI | 0.0000 | 19:0/20:4 PI | 0.0042 |
| 20:1/20:4; 20:0/20:5 PI | 0.0000 | 18:1/20:4 PI | 0.0307 | 20:1/20:5 PI | 0.0013 |
| 20:0/20:4 PI | 0.0002 | 19:1/20:5 PI | 0.0028 | 20:1/20:4; 20:0/20:5 PI | 0.0152 |
| 20:1/20:2 PI | 0.0010 | 19:0/20:4 PI | 0.0093 | 20:0/20:4 PI | 0.0000 |
| 21:1/20:5 PI | 0.0000 | 20:1/20:5 PI | 0.0018 | 20:1/20:2 PI | 0.0000 |
| 21:0/20:5 PI | 0.0000 | 20:1/20:4; 20:0/20:5 PI | 0.0133 | 21:1/20:5 PI | 0.0017 |
| 21:1/20:4 PI | 0.0000 | 20:0/20:4 PI | 0.0089 | 21:0/20:5 PI | 0.0000 |
| 21:0/20:4 PI | 0.0000 | 20:1/20:2 PI | 0.0000 | 21:1/20:4 PI | 0.0000 |
| 22:2/20:5 PI | 0.0000 | 21:0/20:5 PI | 0.0000 | 21:0/20:4 PI | 0.0000 |
| 22:1/20:5 PI | 0.0000 | 21:1/20:4 PI | 0.0002 | 22:2/20:5 PI | 0.0009 |
| 22:1/20:4 PI | 0.0002 | 21:0/20:4 PI | 0.0001 | 22:1/20:5 PI | 0.0006 |

| | | | | | |
|---|---|---|---|---|---|
| 22:0/20:4 PI | 0.0001 | 22:2/20:5 PI | 0.0001 | 22:1/20:4 PI | 0.0012 |
| 22:1/20:2 PI | 0.0103 | 22:1/20:5 PI | 0.0000 | 22:0/20:4 PI | 0.0019 |
| 23:1/20:5 PI | 0.0002 | 22:1/20:2 PI | 0.0079 | 22:1/20:2 PI | 0.0223 |
| 23:1/20:4 PI | 0.0000 | 23:1/20:5 PI | 0.0209 | 23:1/20:5 PI | 0.0239 |
| 23:0/20:4 PI | 0.0099 | 23:1/20:4 PI | 0.0163 | 23:1/20:4 PI | 0.0000 |
| 23:1/20:2 PI | 0.0109 | 23:1/20:2 PI | 0.0018 | 23:0/20:4 PI | 0.0142 |
| 24:3/20:5 PI | 0.0052 | 24:3/20:5 PI | 0.0096 | 23:1/20:2 PI | 0.0032 |
| 24:1/20:5 PI | 0.0000 | 24:1/20:5 PI | 0.0001 | 24:3/20:5 PI | 0.0000 |
| 24:1/20:4 PI | 0.0028 | 24:1/20:4 PI | 0.0163 | 24:1/20:5 PI | 0.0000 |
| 24:0/20:4; 24:1/20:3 PI | 0.0056 | 24:0/20:4; 24:1/20:3 PI | 0.0278 | 24:1/20:4 PI | 0.0083 |
| 24:1/20:2 PI | 0.0380 | 24:1/20:2 PI | 0.0000 | 24:0/20:4; 24:1/20:3 PI | 0.0011 |
| 18:0/20:5 PS | 0.0000 | 18:0/20:5 PS | 0.0011 | 24:1/20:2 PI | 0.0011 |
| 18:1/20:1 PS | 0.0003 | 18:0/20:4 PS | 0.0168 | 18:0/20:5 PS | 0.0000 |
| 20:1/20:5 PS | 0.0000 | 18:1/20:1 PS | 0.0000 | 18:0/20:4 PS | 0.0002 |
| 20:0/20:5; 20:1/20:4 PS | 0.0000 | 20:1/20:5 PS | 0.0000 | 18:1/20:1 PS | 0.0002 |
| 22:1/18:1; 20:1/20:1 PS | 0.0022 | 20:0/20:5; 20:1/20:4 PS | 0.0003 | 18:0/20:1 PS | 0.0440 |
| 21:0/20:5 PS | 0.0003 | 21:0/20:5 PS | 0.0004 | 20:1/20:5 PS | 0.0000 |
| 22:1/20:5 PS | 0.0009 | 23:1/18:1; 22:1/19:1 PS | 0.0028 | 20:0/20:5; 20:1/20:4 PS | 0.0000 |
| 22:0/20:4 PS | 0.0377 | 22:1/20:5 PS | 0.0000 | 22:1/18:1; 20:1/20:1 PS | 0.0187 |
| 23:1/20:1; 22:1/21:1 PS | 0.0003 | 22:0/20:4 PS | 0.0193 | 21:0/20:5 PS | 0.0000 |
| 22:1/22:5 PS | 0.0000 | 42:3 PS | 0.4423 | 21:0/20:4 PS | 0.0303 |
| 22:1/22:2 PS | 0.0299 | 22:1/20:1; 24:1/18:1 PS | 0.0003 | 23:1/18:1; 22:1/19:1 PS | 0.0430 |
| 24:1/20:1; 23:1/21:1 PS | 0.0000 | 23:1/20:5 PS | 0.0000 | 22:1/20:5 PS | 0.0000 |
| 23:1/22:6 PS | 0.0000 | 23:1/20:1; 22:1/21:1 PS | 0.0001 | 22:1/20:4 PS | 0.0004 |
| 24:1/22:6 PS | 0.0482 | 24:1/20:5 PS | 0.0000 | 22:0/20:4 PS | 0.0009 |
| 24:1/22:2 PS | 0.0009 | 22:1/22:5 PS | 0.0016 | 22:1/20:1; 24:1/18:1 PS | 0.0000 |
| Total 99 molecular species | | 24:1/20:4; 22:1/22:4 PS | 0.0419 | 23:1/20:5 PS | 0.0001 |
| | | 24:1/20:1; 23:1/21:1 PS | 0.0037 | 23:1/20:2 PS | 0.0138 |
| | | 23:1/22:6 PS | 0.0002 | 23:1/20:1; 22:1/21:1 PS | 0.0000 |
| | | 23:1/22:5 PS | 0.0158 | 24:1/20:5 PS | 0.0000 |
| | | 24:1/22:6 PS | 0.0019 | 22:1/22:5 PS | 0.0000 |
| | | 24:1/22:4 PS | 0.0053 | 24:1/20:4; 22:1/22:4 PS | 0.0008 |

| | | | | |
|---|---|---|---|---|
| | 24:1/22:2 PS | 0.0179 | 24:1/20:1; 23:1/21:1 PS | 0.0135 |
| | Total 106 molecular species | | 23:1/22:6 PS | 0.0000 |
| | | | 23:1/22:5 PS | 0.0114 |
| | | | 23:1/22:4 PS | 0.0029 |
| | | | 23:1/22:2 PS | 0.0029 |
| | | | 24:1/22:6 PS | 0.0000 |
| | | | 24:1/22:2 PS | 0.0001 |
| | | | Total 112 molecular species | |

[1]*sn*-1 alkyl/ *sn*-2 acyl

[2]*sn*-1(2) acyl/ *sn*-2(1) acyl

PE: Phosphatidylethanolamines; PC: Phosphatidylcholines; PI: Phosphatidylinositols; PS: Phosphatidylserines
